# A Comparative Pulse Accuracy Study of Two Commercially Available Patch Insulin Infusion Pumps

**DOI:** 10.17925/EE.2016.12.02.79

**Published:** 2016-08-28

**Authors:** Jenna L Bowen, Chris J Allender

**Affiliations:** School of Pharmacy and Pharmaceutical Sciences, Cardiff University, Cardiff, UK

**Keywords:** Insulin, patch, pump, pulse, accuracy, subcutaneous insulin infusion

## Abstract

**Background:** Patch pumps are a relatively new method of Insulin delivery. This study explores the accuracy of patch-pumps by reporting on comparative pulse-accuracy study of two patch pumps. **Methods:** The accuracy of two patch pumps (Cellnovo, [Cellnovo Ltd., Swansea, UK] and OmniPod^®^ [Ypsomed Ltd, Escrick, UK]) was evaluated micro-gravimetrically. Pulse accuracy was analysed by comparing single and time-averaged pulses for each device. **Results:** Single-pulses outside accuracy thresholds ±5%, ±10%, ±15%, ±20%, ±25% and ±30% were: Cellnovo; 79.6%, 55.6%, 35.0%, 19.9%, 9.7% and 4.3%; OmniPod; 86.2%, 71.6%, 57.4%, 45.5%, 35.2% and 25.4%. For 10, 20 and 40 pulse-windows mean values outside ±15% accuracy level were: Cellnovo; 7.3%, 1.5% and 0.4%, OmniPod; 37.6%, 31.8% and 25.9. **Conclusions:** This study showed that not all patch pumps are the same. The pumping mechanisms employed in these pumps play a significant role in the accuracy and precision of such devices.

Insulin pumps provide a convenient way of delivering a continuous subcutaneous insulin infusion (CSII). Devices are highly flexible to individual patient needs, allowing personalised 24-hour basal infusions and on-demand bolus delivery for acute periods of elevated blood glucose (for example, carbohydrate consumption during meals). Insulin pumps offer patients certain advantages over multiple daily injection (MDI) regimens including fewer injections, more flexibility and the ability to better fine-tune regimens to their personal lifestyle/needs.^1^ Clinically, they have been shown in randomised, controlled trials to provide improved glycaemic control (lower glycated haemoglobin [HbA_1c_])^2,3^ reduce the frequency of hypoglycaemic episodes,^3,4^ and enhance quality of life versus specific MDI regimens in type 1 diabetes mellitus.^2^ For the paediatric population, quality of life gains, extend beyond pump users to their families and carers.^5^

Several varieties of insulin pump are commercially available, with the two main products being durable pumps and patch pumps. Durable pumps are the most common and include an infusion set that connects the subcutaneous cannula to the pump device via an infusion line (~30-100 cm). Examples include the Animas Vibe^®^ and the Animas Ping^®^ (Animas, West Chester, Pennsylvania, US), the Accu-Chek^®^ Combo (Roche, Basel, Switzerland), the MiniMed Paradigm^®^Veo™ (Medtronic, Dublin, Republic of Ireland) and the DANA Diabecare R^®^ (Advanced Therapeutics, Sooil, Seoul, Korea).

Unlike durable pumps, patch pumps are free of infusion sets as the cannula and delivery system are built into the device. They are worn directly on the body and controlled by a wireless device making them more discrete than the traditional durable pumps. Patch pumps aim to increase patient compliance by providing freedom from long-tubing, increased flexibility, easier technical operation and a smaller, lightweight device capable of being manipulated discretely.^6^ Examples include the OmniPod^®^ (Insulet Corporation, Billerica, Massachusetts, US) and the Cellnovo system (Cellnovo Ltd., Swansea, UK).

Whilst patch pumps offer clear aesthetic advantages, a recent study found the dosing accuracy of the OmniPod patch pump to be unfavourable when compared to several durable pumps.^7^ Jahn et al.^7^ demonstrated that the patch pump was significantly less accurate in terms of both single-pulse and averaged-pulse accuracy, than the traditional durable pumps. Unfortunately, only one patch pump (OmniPod) was investigated versus three durable pumps (OneTouch Ping, Accu-Chek Combo and the MiniMed Paradigm Revel™/ Veo). In another study, Cappuro et al.^8^ sought to compare the dose precision performance of the Animas Vibe and t:slim^®^ (Tandem^®^ Diabetes Care, San Diego, California, US) durable pumps and the OmniPod patch pump over three delivery phases in a 20 hour test. Results showed that across all delivery stages and in terms of dose variability, the OmniPod did not perform as well as the Animas Vibe.

**Figure 1:**
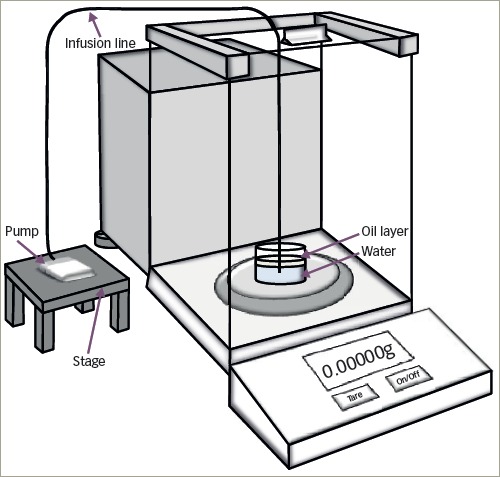
Schematic of the test apparatus used to investigate patch pump accuracy

A number of methods are available to compare pumps, including those referenced in the Worldwide Standard EN 60601-2-24:2012, methods using pipettes, microscopes and imaging software^13^ as well as the method described by Jahn et al.^7^

To date no groups have sought to compare dosing accuracy between commercially available patch pumps. We report a comparative pulse accuracy study comparing OmniPod and Cellnovo patch pump devices. The Cellnovo system utilises a post-reservoir, wax-powered micro-pump and micro-fluidics that dispenses insulin on demand in a pulsatile fashion. It has a closed-loop feedback mechanism that continually interrogates a reservoir position sensor residing within the durable element of the pump. It has the ability to make drop-by-drop alterations to the delivered volume to ensure continuous, accurate delivery. By contrast, the OmniPod system employs a more traditional disposable syringe pump mechanism to actuate movement of its reservoir plunger, relying on a shape-memory alloy to drive its motor, utilising a paired tick-tock action. The Cellnovo system is approved for use within the EU through its CE Mark, the OmniPod system is available for use within the US as well as the EU and other countries.

The Methodology employed in this study was a slightly modified version of that described by Jahn et al.^7^ Specific adjustments were made to the setup to account for comments made by Zisser^12^ regarding the potential for mechanical oscillations to create measurement spikes when housing the patch pumps within the measurement equipment. Pumps were compared by evaluating single and average-pulse accuracy over clinically relevant periods of pump use. It is important to note that this was not intended to be a clinical study, merely one that evaluates relative technical performance of two patch pump systems.

## Materials and methods

The accuracy of the OmniPod and Cellnovo patch pump devices was investigated using a modified version of the time-stamped micro-gravimetric system reported by Jahn et al.^7^ (*Figure 1*). The system comprised two Discovery DV215CD semi-micro analytical balances (81 g capacity, 0.00001 g resolution, Ohaus, Nanikon, Switzerland) positioned on a robust low-vibration table. Balances were internally calibrated before use and all measurements performed at room temperature with the balance draft-shield doors closed. Balance data was captured at 90-second intervals using Quod Pump Controller V6.1 software (Cellnovo Ltd., Swansea, UK). The Quod Pump Controller software is designed to record balance data at predetermined time intervals. The software was set to capture data at a frequency greater than the delivery frequency.

A circular plastic vial (diameter 4 cm, capacity 25 ml) was filled with 15 ml deionised water and placed on the weighing pan of the balance. A thin layer of paraffin oil (1.5 ml) was applied to the top of the water. This volume of oil sufficiently covered the surface of the water, minimising evaporation during experimental runs. Patch pump devices were positioned outside of the balance and connected to the pre-filled plastic vial via clear, flexible infusion lines (60 cm length, 0.8 mm i.d., 2.4 mm o.d., TYGON R-3603 laboratory tubing [Fisher Scientific UK Ltd., Leicestershire, UK]) of similar diameter to the devices’ cannula (OmniPod) or connector (Cellnovo). For the OmniPod device, the infusion line was sealed around the base of the cannula using UV-activated resin (BUG-BOND™). To ensure that the OmniPod was connected appropriately and running properly, discrete bolus pulses were delivered to prime the infusion line and to ensure, through careful observation, that the system was leak and obstruction free. A 60 cm length was chosen to ensure that the tubing was of a reasonable kinkfree length from the patch pumps to the weighing system. The Cellnovo device came fitted with an outlet valve connector that was used to connect the device to the infusion line. Within the balance ‘cabinet’ the infusion line was fitted with a needle (length, 3.5 cm) the end of which was positioned through the paraffin oil layer to project (~2 mm) into the underlying water. The needle was projected into the underlying water to reduce any evaporation effects and to ensure that each drop was fully assimilated into the underlying water in a way that the increased mass could be measured. To reduce the effects of siphoning, the patch pump devices were positioned on stages so that the device output valve/cannula was level with the tip of the needle in the collecting vial.

As in Jahn et al.,^7^ de-gassed deionised water was used as a surrogate neutral infusion fluid for fast-acting insulins giving comparable fluid properties. This complies with the international standard set out in EN 60601-2-24:2012 that with regard to the testing of essential performance of infusion pumps and controllers, requires the use of a liquid which can be expected to give similar test results to the liquid intended for use.^9^

Both devices were loaded with deionised water primed and programmed as specified by the manufacturer’s instructions. As both pumps deliver fixed 0.5 μl volumes, the rate of insulin delivery was controlled by varying the number of pulses per hour.^10^ For both pumps and for all runs, the initial pulse rate was set to 0 μl/hour for 1 hour in order to gauge system stability and measure weight-loss due to evaporation. All pumps were then run for 200 pulses per run. Evaporation and system stability was again, evaluated at a pulse rate of 0 μl/hour for one hour immediately post the 200 pulse run period (*Table 1*). The total run time was therefore 22 hours with weights recorded every 90 seconds (two measurements for each delivery point) to ensure delivery was measured correctly at each point.

A total of 30 runs were completed for each device (n=30, two repetitions for each of 15 different pumps). Each pump was limited to two repeats due to the 72-hour expiry of the pumps after priming. Comparisons between pulse data of the devices were made for the 20-hour test basal rate period.

**Table 1:** Run protocol and description to evaluate system stability and patch pump reliability

Run period	Pulse rate (hour-^1^)	Description
Hours 0–1	0.0	Pre-run stability check
Hours 1–21	10–20	Test basal rate
Hours 21–22	0.0	Post-run stability check

## Data analysis

Data for each individual 0.5 μl pulse were isolated, and recorded. Pulse volume was derived directly from pulse weight (∆W, simply the weight difference recorded by the balance between discrete pulse) using equation 1. The percentage error in pulse volume was then calculated according to equation 2.

[1] ΔW(μg)998.21 (μg H2O ml−1)= pulse delivered (μl)

[2] $$\frac{\mathrm{Volume pulse delivered-Volume pulse expected}}{\mathrm{Volume pulse expected}}\;\times \;100\;=\;\%\mathrm{Error in pulse volume}$$

### Single-pulse accuracy

For both devices, single-pulse accuracy (percentage deviation from expected pulse volume) was analysed for each discrete pulse delivered over the 30 runs (n=6000 pulses) according to equation 2. Using these data the number of discrete pulses with percentage error greater than predetermined accuracy thresholds (±5, 10, 15, 20, 25 and 30% deviation from expected pulse volume) was calculated and compared for the two patch pump devices.

### Averaged pulse accuracy

Although investigating single-pulse accuracy is a valid metric to assess pump performance, averaged-pulse accuracy over sustained periods of pump delivery may be a more clinically relevant assessment since patch pumps are used continuously and the time to reach steady-state will vary. Averaged pulse accuracy was analysed by averaging discrete pulse errors over predetermined observation windows. For example, at a dosing rate of 1 unit per hour, 10, 20 and 40 consecutive pulse errors were averaged to calculate the averaged-pulse accuracy over a 0.5 units, 1 unit and 2 units respectively.

### Typical patch pump performance

To further compare the two pumps, and specifically to gain insight into the underlying pumping mechanisms, a ‘typical performer’ was selected from the 15 individual devices tested for both OmniPod and Cellnovo. The typical pump was selected as the pump that had the median standard deviation in discrete pulse error over the 20-hour test basal rate period.^7^

### Statistical analysis

Unpaired t-tests were used for all comparisons between the devices. The standard deviation of discrete dose percentage errors was calculated for each of the 30 experimental runs for both devices.

## Results

### Single-pulse accuracy

**Figure 2:**
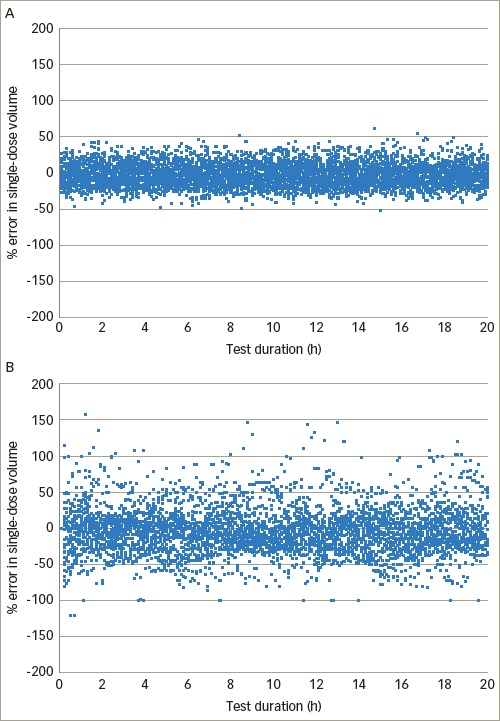
Overall patch pump performance showing percentage error in single-pulse volume for the Cellnovo (A) and OmniPod (B) devices (n=6000 discrete pulses)

Investigating the accuracy of discrete pulses is one way of assessing the performance of insulin infusion pumps. *Figure 2* shows the percentage error in single-pulse volume for each pulse delivered over the 30 experimental runs for the Cellnovo (*Figure 2A*) and OmniPod (*Figure 2B*) devices. Single-pulse accuracy ranged from -120.0% to 158.5% for the OmniPod pump and -51.6% to 61.8% for the Cellnovo pump.

**Figure 3:**
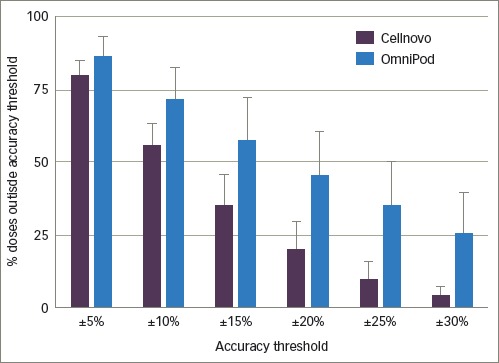
The mean percentage of single pulses outside of the ±5, 10, 15, 20, 25 and 30% accuracy thresholds for the OmniPod and Cellnovo patch pumps over the 20-hour basal rate period The percentage of delivered pulses outside of the accuracy thresholds was significantly lower for the Cellnovo pump at all thresholds (p<0.0001, n=30 runs ± standard deviation).

The percentage of single pulses delivered outside accuracy thresholds of ±5%, ±10%, ±15%, ±20%, ±25% and ±30% were: Cellnovo 79.6%, 55.6%, 35.0%, 19.9%, 9.7% and 4.3%; OmniPod 86.2%, 71.6%, 57.4%, 46.5%, 35.2% and 25.4%) (*Figure 3*, *Table 2*). There is a significant difference between Cellnovo and OmniPod value for all of these thresholds (p<0.0001).

### Averaged pulse accuracy

**Figure 4:**
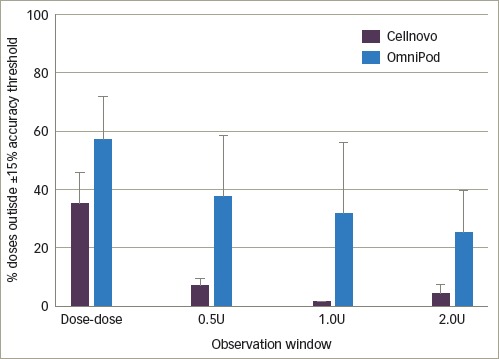
The mean percentage of pulses outside of the ±15% accuracy threshold averaged over observation windows of 0.5, 1.0 and 2.0 units for the OmniPod and Cellnovo patch pumps over the 20-hour basal rate period Dose-dose refers to single, non-averaged pulses. The percentage of delivered pulses outside of the accuracy threshold was significantly lower for the Cellnovo pump for all observation windows (p<0.0001, n=30 runs ± standard deviation). U=unit

An alternative and perhaps more clinically relevant way to assess patch pump performance is to investigate averaged pulse accuracy over extended periods of delivery. The averaged pulse accuracy of the pumps was investigated over pre-determined observation windows of 10, 20 and 40 pulses (nominally 0.5, 1.0 and 2.0 units) (*Figure 4*, *Table 2*). For both pumps the averaged-pulse accuracy improved as the observation window increased. The percentage of pulses delivered outside of the ±15% accuracy threshold over 0.5 unit, 1.0 unit, and 2.0 unit observation windows, were: Cellnovo 7.3%, 1.5% and 0.4%; OmniPod 37.6%, 31.8% and 25.9% respectively (*Figure 4*). There is a significant difference between Cellnovo and OmniPod value for all of these thresholds (p<0.0001).

### Typical pump performance

**Figure 5:**
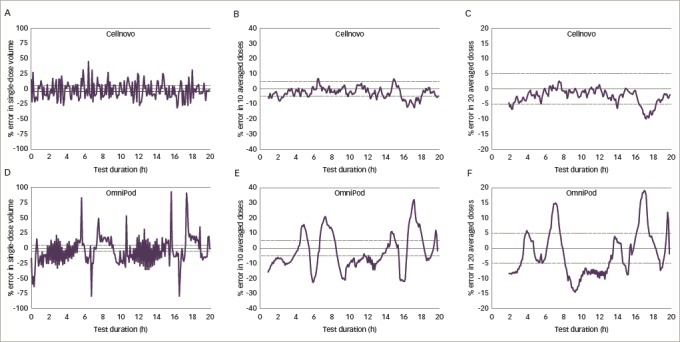
20-hour basal profiles of a typical Cellnovo (A-C) and OmniPod (D-F) patch pump showing single-pulse accuracy (AD), 10-pulse averaged accuracy (B,E) and 20-pulse averaged accuracy (C,F). For all figures the dotted horizontal lines indicate the ±5% accuracy range.

The performance of a typical pump, selected as the pump that exhibited the median standard deviation in discrete pulse error, was investigated by plotting the single-pulse accuracy (*Figure 5A* and *D*), 10-pulse averaged accuracy (*Figure 5B and E*) and 20-pulse averaged accuracy (*Figure 5C* and *F*) of the device over the entire 20-hour test basal rate period. The Cellnovo pump exhibited markedly less variability in pulse accuracy (*Figure 5A*-*C*) than the OmniPod pump (*Figure 5D*-*F*). When dosing accuracy was averaged over a 2-hour time period the OmniPod profile still exhibited a highly variable dosing profile (*Figure 5F*).

### Industry-established flow accuracy

**Figure 6:**
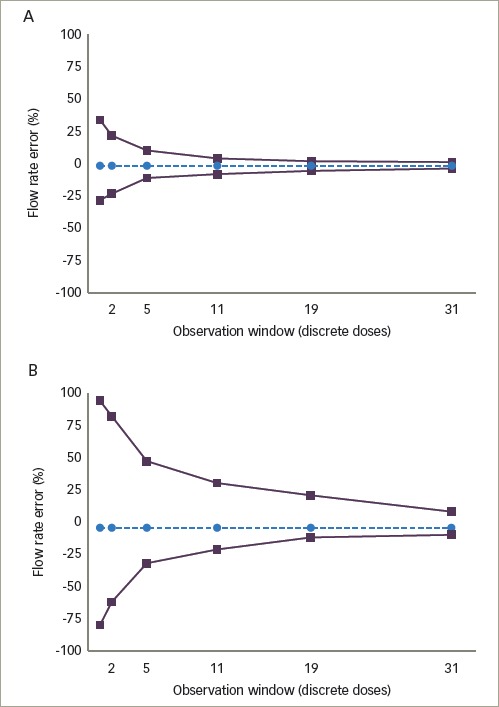
Trumpet curves for a typical Cellnovo and OmniPod patch pump device Cellnovo (A) and OmniPod (B) patch pump device showing the maximum positive and maximum negative flow rate error (black solid lines) and the average flow rate error (black dotted line) for 2, 5, 11, 19 and 31 pulses.

Pump flow accuracy over the last 100 deliveries in the 20-hour test basal period was compared for a typical Cellnovo and OmniPod pump as outlined in EN 60601-2-24:2012. Maximum positive and negative percentage deviations across a 2, 5, 11, 19 and 31 pulse window were calculated (*Figure 6*). For both pumps the stabilisation period prior to the last 100 delivery assessment was 10 hours.

**Table 2:** Single and averaged-pulse accuracy of the two patch pump devices. Data shows the mean percentage of pulses delivered outside of single and averaged-pulse accuracy thresholds (±5-30%)

Dosing Accuracy over 20 hours		
	% outside accuracy threshold (6000 pulses)	
	Cellnovo	OmniPod®
Single Dose (±)		
5%	79.6	86.2
10%	55.6	71.6
15%	35.0	57.4
20%	19.9	45.5
25%	9.7	35.2
30%	4.3	25.4
0.5 Unit averaging window (±)		
5%	51.5	76.9
10%	21.8	55.4
15%	7.3	37.6
20%	1.9	22.1
25%	0.7	13.1
30%	0.5	7.8
1 Unit averaging window (±)		
5%	42.2	71.9
10%	10.9	48.5
15%	1.5	31.8
20%	0.2	17.6
25%	0.0	9.1
30%	0.0	5.3
2 Unit averaging window (±)		
5%	37.9	66.3
10%	7.2	41.0
15%	0.4	25.9
20%	0.0	14.1
25%	0.0	7.7
30%	0.0	3.9

## Discussion

Patch insulin infusion pumps are a relatively recent innovation aiming to increase CSII compliance for insulin-dependent diabetes patients. Whilst offering advantages in the form of discreteness, ease of use and overall patient satisfaction, a recent study found the dosing accuracy of a patch pump to be unfavourable compared to the traditional durable pumps;^7^ it was significantly less accurate in terms of both single and averaged-pulse measurements.

The current technical evaluation has demonstrated that the Cellnovo device displayed significantly better single-pulse accuracy than the OmniPod pump when assessed over predetermined accuracy thresholds (±5–30%), and was significantly more accurate when assessed over longer, more clinically relevant observation doses (0.5–2 units).

The markedly different performance may be explained by the differing pumping mechanisms integral to these devices. Any shortcomings found in the delivery performance of the OmniPod system can be attributed to the disposable nature of the device. The results reflect those of Borot et al.,^11^, who used a similar method to Jahn et al.,^7^ excepting that they used the same method to test both types of pumps and as with the current study, placed the pumps outside the microbalance. Results demonstrated that *in vitro*, the patch pump studied was more accurate than the comparators, including the Omnipod.

## Limitations

There have been discussions in the literature on the optimal manner in which insulin pump delivery should be measured for precision and accuracy.^7,11,12,13^ Jahn et al.^7^ discuss a methodology that compares a reduced number of durable systems (n=6) in comparison to the patch pump (n=15) and this could be seen as an inconsistency, leading to greater variability being witnessed in the larger sample number. However, in normal use, a patient would be wearing a single durable pump for four years and would change their patch pump every three days; this increases the need to understand the actions of disposable pumps both throughout their life and understanding the pump to pump (or pod to pod variability). It is believed that this information would have led to the increased *n* in the patch pump arm of the study, and also made fairer by the use of multiple durable pumps. The Cellnovo system in this test consists of a durable element and a 3-day disposable element; it was however, tested by the same means as the disposable system.

The experimental design of the current evaluation mirrored that of Jahn et al.^7^ with slight modifications, notably the infusion setup. This was for two reasons: first, while this study did not seek to compare testing methodologies, the authors took into account the comments made by Zisser^12^ about dose-to-dose delivery accuracy. Zisser^12^ suggested that in Jahn et al.’s. study,^7^ the positioning of the Omnipod within the microbalance may have been the reason for the measured oscillatory data seen in the Omnipod data. In this study this was taken into consideration and both patch pumps were positioned outside of the microbalance and connected via a tube to ensure a consistent and fair test; second, in the Jahn et al. study, durable pumps were attached to infusion lines whereas the OmniPod pump was attached to the collecting vial directly by the device cannula. This was done to mirror the clinical situation, as durable pumps require infusion lines whereas patch pumps do not. However, this comes at the expense of introducing experimental design difference between the groups. Furthermore, we found that the short length of the cannula (6.5 mm for OmniPod, 5.0 mm for Cellnovo system) made it difficult to connect directly to the collecting vial whilst achieving a steady system setup. In this study both pumps were connected to the collecting vial by an infusion line ensuring both pumps were compared using identical experimental setups. Although this is a potential limitation of the study, as in practice patch pumps are free from infusion lines, it ensured a consistent and fair comparison between the pumps.

As mentioned previously there are alternative methods for measuring pulsed dose accuracy.^13^ These methods are not easily employable for the measurement of large numbers of systems and care must be taken when measuring spheres with such small radii due to evaporation rates in varying environmental humilities.

## Conclusions

Whilst a previous study showed that a patch pump performed poorly when compared to a number of durable pumps, this study showed that not all patch pumps are the same. The pumping mechanisms employed in these pumps play a significant role in the accuracy and precision of such devices, which in turn may impact on clinical outcome.
